# Targeting Autophagy in ALK-Associated Cancers

**DOI:** 10.3390/cancers9120161

**Published:** 2017-11-27

**Authors:** Julie Frentzel, Domenico Sorrentino, Sylvie Giuriato

**Affiliations:** 1 Merck Serono S.A., Route de Fenil 25, Z.I. B, 1804 Corsier-sur-Vevey, Switzerland; julie.frentzel@gmail.com; 2 Inserm, UMR1037, CNRS, ERL5294, Université Toulouse III-Paul Sabatier, CRCT, F-31000 Toulouse, France, domenico.sorrentino@inserm.fr; 3 European Research Initiative on ALK-related malignancies (ERIA); 4 TRANSAUTOPHAGY: European Network for Multidisciplinary Research and Translation of Autophagy Knowledge, COST Action CA15138

**Keywords:** ALK (anaplastic lymphoma kinase) oncogene, anaplastic large cell lymphoma (ALCL), non-small cell lung carcinoma (NSCLC), neuroblastoma (NB), rhabdomyosarcoma (RMS), tyrosine kinase inhibitor (TKI), combined therapy, cytoprotective autophagy, cytotoxic autophagy, autophagic switch

## Abstract

Autophagy is an evolutionarily conserved catabolic process, which is used by the cells for cytoplasmic quality control. This process is induced following different kinds of stresses e.g., metabolic, environmental, or therapeutic, and acts, in this framework, as a cell survival mechanism. However, under certain circumstances, autophagy has been associated with cell death. This duality has been extensively reported in solid and hematological cancers, and has been observed during both tumor development and cancer therapy. As autophagy plays a critical role at the crossroads between cell survival and cell death, its involvement and therapeutic modulation (either activation or inhibition) are currently intensively studied in cancer biology, to improve treatments and patient outcomes. Over the last few years, studies have demonstrated the occurrence of autophagy in different Anaplastic Lymphoma Kinase (ALK)-associated cancers, notably ALK-positive anaplastic large cell lymphoma (ALCL), non-small cell lung carcinoma (NSCLC), Neuroblastoma (NB), and Rhabdomyosarcoma (RMS). In this review, we will first briefly describe the autophagic process and how it can lead to opposite outcomes in anti-cancer therapies, and we will then focus on what is currently known regarding autophagy in ALK-associated cancers.

## 1. Introduction

The Anaplastic Lymphoma Kinase (ALK) is physiologically expressed in a specific subset of neuronal cells during the embryonic life [1]. Its aberrant expression and activation, following diverse gene rearrangements (amplification, mutations, or chromosomal translocations) are responsible for the development of a large spectrum of cancers [2,3]. ALK contains a tyrosine kinase domain, whose constitutive and unrestrained activation, in the oncogenic forms of the protein, accounts for its tumorigenic potential [2,4]. Therefore, over the past ten years, and driven by the impulsion of the success of breakpoint cluster region-abelson (BCR-ABL) tyrosine kinase targeted therapies that are developed for Chronic Myelogenous Leukemia patients [5,6], researchers and clinicians have worked in concert for the development of specific ALK tyrosine kinase targeted therapies. As a successful result of such a mobilization, an arsenal of ALK tyrosine kinase inhibitors (ALK TKIs) is now available [3,7,8,9], and their use has been proven to be beneficial in many ALK-associated cancer patients [10,11,12]. However, as a known feature following targeted therapies, ALK-positive cancer cells were found to evolve and to develop resistance mechanisms against those TKIs [13,14,15,16]. To counteract these resistances, the combination of ALK TKIs, with other drugs, targeting the survival pathways that are involved in the acquisition of resistance, appeared logically as an appealing approach to eradicate cancer cells [17,18]. 

In this framework, autophagy has had a burst of interest over the past ten years, especially in the cancer research field [19]. Depending on the context, autophagy has been shown to harbor a dual role following cancer therapy, acting either as a tumor cell survival mechanism, or, oppositely, as a tumor cell death-associated process [20,21]. Thus, the therapeutic modulation of autophagy in cancer is an ongoing important research field. In this review, we will present the autophagic process, its classical measurements, and its functions in cancer therapy. Then, we will discuss the current knowledge about its role in the treatment of ALK-associated cancers, especially in ALK-positive Anaplastic Large Cell Lymphoma (ALCL), ALK-positive Non-Small Cell Lung Carcinoma (NSCLC), Neuroblastoma (NB) and Rhabdomyosarcoma (RMS).

## 2. Autophagy: Definition and Measurements

### 2.1. History of Autophagy

The word “autophagy” originates from the greek words “*auto*” meaning “self”, and “*phagein*”, meaning “to eat”. It was used for the first time in the early 1960s by Pr Christian De Duve who won the Nobel Prize in Medicine in 1974 for the discovery of the lysosome, the functional organelle of the autophagic process [22]. From this date, several other groups accumulated evidences allowing for the characterization of autophagy [23,24]. These efforts ultimately lead to the identification of all the genes regulating the process by Pr Yoshinori Ohsumi [25,26], for which he was awarded the Nobel Prize in physiology or medicine in 2016. The interest of the scientific community towards this process has rapidly been expanding so that in 2010, more than 45 papers a week were published on the subject.

### 2.2. The Autophagic Process

**Figure 1:**
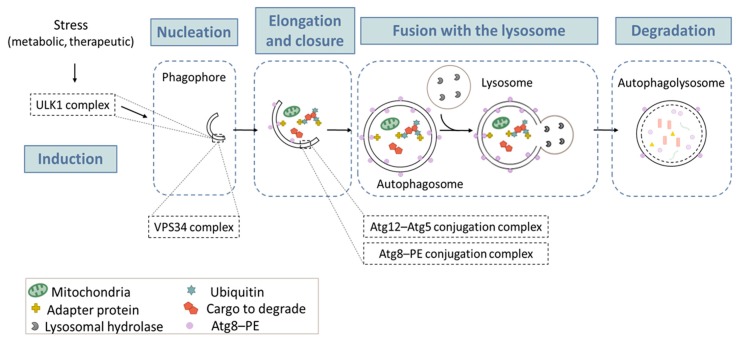
**The macroautophagy process.** Macroautophagy takes place in five main steps: (1) Induction occurs after a metabolic or therapeutic stress, and is mediated by a complex containing the ULK1 protein. (2) During the nucleation step, the formation of the phagophore (or isolation membrane) is initiated. This action is mainly triggered by a protein complex containing VPS34, a PtdIns3K of class III. (3) Elongation of the phagophore involves two «ubiquitin like» conjugation systems: the Atg12–Atg15 and the Atg8–PE, which are required for autophagosome formation. (4) Once formed, the autophagosome containing a cytosolic cargo will fuse with the lysosome, which triggers (5) the degradation of its content and the release of primary components in the cytosol for recycling. The lysosome can then be regenerated so that the process can start again.

Autophagy is a physiological catabolic process that is highly conserved in all eukaryotes. Its main function is to regulate homeostasis within the cell by allowing nutrient recycling. Currently, three types of autophagy have been described: macroautophagy, microautophagy and chaperone-mediated autophagy (CMA) [27]. The most studied type is macroautophagy, herein referred to as autophagy [28]. Autophagy is hyper-activated under different stimuli, including nutrient deprivation, metabolic stress, anticancer drugs, and radiation. As illustrated in Figure 1, autophagy begins with a small part of the cytoplasm that is sequestered in a membrane sac, also called “phagophore”. The elongation and completion of the phagophore generate a double-membrane structure, called the “autophagosome”, which is a unique organelle, both in its structure and its dynamic regulation. The process ends with docking and fusion between the autophagosome and the lysosome, forming an autophagolysosome. Following fusion, lysosomal enzymes degrade the enclosed cytoplasmic materials. This degradation results in the recycling of amino acids and other products, for later use by the cell [29]. The autophagic process has been divided into five successive steps (initiation, nucleation, elongation, fusion, and degradation) (Figure 1), which are tightly orchestrated by key proteins, including autophagy-related (ATG) proteins, and several multi-proteic complexes, such as the complex ULK1 (unc-51-like kinase 1) in the initiation phase; the class III PtdIns3K (phosphatidylinositol 3-kinase) (best known as VPS34) complex, in the nucleation phase; and the two ubiquitin-like conjugation systems in the elongation phase (one responsible for ATG12–ATG5 conjugation, and the other one allowing for the maturation of the mammalian orthologs of the yeast Atg8 protein). Detailed descriptions of the autophagy machinery could be found in excellent reviews [28,30,31]. Below, we will only focus on the ATG8 conjugation system (paragraph “Turnover of PE-conjugated ATG8 proteins”), since it is widely used to monitor autophagy. 

### 2.3. Classical Measure of Autophagy

Guidelines to monitor autophagy are published and are frequently updated [32]. In this section, we will highlight a few common methods that are used to monitor autophagy. Usually, it is recommended to combine at least two or three of these methods to obtain a reliable measurement.

#### 2.3.1. Transmission Electron Microscopy (TEM)

TEM was the first technique that was used to detect autophagy. It is the only autophagy monitoring technique enabling the visualization of autophagic structures and their position within the cell. Although TEM remains the traditional method that is used in the field, it requires high technical expertise to analyze the obtained pictures and to identify organelles and autophagosomes in particular. It is also very time consuming, and a special care has to be taken to guarantee proper handling of the samples.

#### 2.3.2. Labelling of Acidic Vesicules

Cell staining with acidotropic dyes, such as Acridine Orange and Neutral Red, is also used as an indicator of autophagy. These dyes are not ideal markers for this process because they stain all acidic vesicles that are present in cells, including lysosomes. Although not specific, these dyes can be informative when combined with other techniques allowing for autophagy monitoring.

#### 2.3.3. Turnover of PE-Conjugated ATG8 Proteins

MAP1LC3 (microtubule associated protein 1 light chain 3), best known as LC3, belongs to the ATG8 family of proteins. This family is divided into two subgroups, i.e., the LC3 and the GABARAP (gamma-aminobutyric acid receptor-associated protein) proteins. Four LC3 isoforms (LC3A, B, B2 and C) and three GABARAP isoforms (GABARAP, GABARAPL1 and GABARAPL2/GATE-16 (Golgi-associated ATPase enhancer of 16 kDa)) have been identified in mammals. The maturation of these ATG8 proteins involves the cleavage of their precursor by a cysteine protease (ATG4), and their subsequent lipidation (addition of a phosphatidylethanolamine molecule (PE)). The non-lipidated forms are referred to LC3-I or GABARAP-I, and the lipidated forms (which are the ones that are associated with the autophagosomal membranes) are referred to LC3-II or GABARAP-II. In the literature, the induction of autophagic flux is traditionally evaluated by observing the difference by western blot in the amount of LC3B-II in both the presence and absence of lysosomal inhibitors, such as bafilomycin A1 and chloroquine. If autophagy is induced, the amount of LC3B-II will be higher in the presence of the inhibitor than in its absence. The same reasoning applies for GABARAP-I and GABARAP-II. 

It should be noted that when using this technique, the researchers are limited by the specificity of the antibodies that are commercially available. For instance, cross-reactions between LC3A and LC3B, as well as between GABARAP and GABARAPL1, are known and can lead to result misinterpretation. 

Regarding GABARAP as a marker for autophagy measurement, it is important to point out that the PE-conjugated GABARAP forms are usually undetectable in mammalian cells without autophagy induction [33]. In addition, autophagy was found to be LC3 independent in certain cell types. In those cases, GABARAP is absolutely required to fulfill the autophagy process [34]. Because of these particularities, it has been proposed that this subfamily of protein might be more sensitive than the LC3 family to monitor autophagy induction [32]. However, for now, LC3B remains the most common autophagy marker used in the literature.

#### 2.3.4. Fluorescent LC3B Probes

Techniques using fluorescence probes to monitor autophagy are also frequently used. As an example, autophagy induction can be monitored by the ectopic expression of GFP (green fluorescent protein)-LC3 in cells. During autophagy induction, the cytoplasmic GFP-LC3 protein will relocate to the autophagosomes, inducing the formation of fluorescent puncta, which can be visualized and quantified by fluorescent microscopy. This technique is more sensitive than the monitoring of LC3B-II by western blotting, but it often requires some technical optimization rounds. The autophagic flux can also be evaluated with an RFP (red fluorescent protein)-GFP-LC3 tandem construct [35]. While autophagosomes bearing this fusion protein will appear as yellow dots (i.e., both RFP and GFP positive), the autophagolysosomes will appear in red (RFP), as the GFP fluorescence is quenched upon lysosomal acidification (pH < 5). The autophagic flux induction can thus be quantified by the loss of the GFP fluorescence. This characteristic can also be measured by using a flow cytometer, and allows for the ratiometric quantification of autophagy induction [36].

The critical point for an accurate quantification of autophagosomes and autophagolysosomes, and for a correct assessment of the autophagic flux with these fluorescent probes, is the sensitivity of the green fluorescent protein to acidic pH. Thus, after the initial development of the RFP-GFP tagged LC3B construct [35], other groups have generated refined versions of this probe, replacing GFP with mWasabi [37] or pHluorin [38], which appeared to be more suitable to precisely monitor autophagic structures. 

Recently, a new autophagic flux probe, i.e., GFP-LC3-RFP-LC3∆G, has been developed by the group of Pr Mizushima [39,40]. It is based on the equimolar release of GFP-LC3 and RFP-LC3∆G upon cleavage by endogenous ATG4 proteases. The RFP-LC3∆G fusion protein does not contain the C-terminal glycine residue mandatory for lipidation, it will thus stay free in the cytosol and serves as an internal control. On the contrary, GFP-LC3 can relocate to the autophagosomes, and after its fusion with lysosomes, the fluorescence signal will ultimately be lost. The measured GFP:RFP signal ratio inversely correlates with autophagy flux activation. This system appears to be more sensitive than the classical RFP-GFP-LC3B probe because it avoids the late degradation of RFP within autophagolysosomes.

#### 2.3.5. Autophagic Cargo Sequestration Assay

This assay allows for the measurement of functional autophagy, i.e., the effective sequestration of an endogenous proteic cargo from the cytosol to the autophagosomes [41]. Initially developed in 1990 [42], this method has been optimized using LDH (lactate dehydrogenase) as a cargo marker. This protein offers the multiple advantages to be ubiquitously expressed; to be quantitatively detected through its enzymatic activity (measured as the decrease in NADH (Nicotinamide Adenine Dinucleotide Hydrogen) absorbance); and, to be exclusively degraded by autophagy. Experimentally, this also called “LDH sequestration assay” is based on the separation and subsequent quantification of soluble (cytosolic) and sequestered LDH (sedimented autophagic vacuoles) [43].

## 3. Autophagy in Cancer Therapy

### 3.1. Cytoprotective or Cytotoxic Functions of Autophagy Following Cancer therapy

Autophagy activation following cancer therapies has been associated mainly with either cancer cell survival or cancer cell death [44,45]. A small number of studies also report a cytostatic and a non-protective function for autophagy, but these two responses have been less studied thus far [46,47,48,49]. The cellular and molecular mechanisms underlying the two main and opposite outcomes in the tumor cells’ fate (survival or death) following autophagy activation are not clearly understood yet. 

So far, it has been proposed that the mechanisms explaining the cytoprotective function of autophagy following therapy mainly rely on the impairment of the apoptotic cell death pathway. This can involve (i) the clearance of drug-induced cytotoxic reactive oxygen species (ROS) [50]; and, (ii) the degradation of pro-apoptotic proteins [51]. Additionally and importantly, cytoprotective autophagy has been shown also to protect cancer stem cells through the induction of tumor dormancy [52,53].

Conversely, the cytotoxic function of autophagy has been described to mainly rely on the promotion of diverse cell death mechanisms. This can involve (i) the induction of apoptosis through the activation of key apoptosis protein [54,55]; (ii) the degradation of negative regulator of apoptosis [56,57]; (iii) the use of elongating autophagic membranes as a scaffold for the assembly of the apoptosis protein complex (called apoptosome) [55]; (iv) the induction of necroptosis [58]; and, (v) the induction of immunogenic cell death [59,60]. Notably, autophagy on its own can also lead to cell death through an excessive and lethal self-digestion [61].

### 3.2. Autophagic Switch

**Figure 2:**
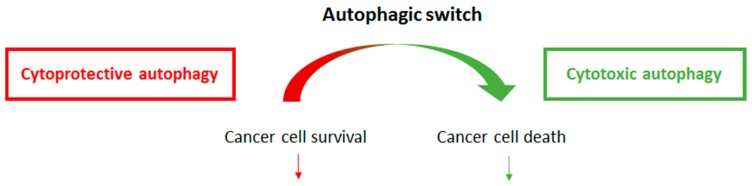
**Autophagic switch from a cytoprotective to a cytotoxic function.** Cytoprotective autophagy prevents the cancer cell death and is induced by some particular conditions in various cancer models. All the conditions leading to cytoprotective autophagy are colored in red. When an autophagic switch occurs, cytoprotective autophagy turns into cytotoxic autophagy, and helps to kill the cancer cells. All the conditions leading to the induction of cytotoxic autophagy are colored in green.

Adding complexity in identifying the function of autophagy following cancer therapy, a growing amount of studies are pointing to the possibility for a singular cancer cell to undergo a shift from cytoprotective to cytotoxic autophagy. This switch is beginning to be understood at a molecular level, and usually relies on an additional signaling partner (or pathway), which modifies the autophagy magnitude within the treated cells. Examples of autophagic switches are reported in Figure 2.

A series of studies carried out in breast tumor cells demonstrated the switch from cytoprotective autophagy in cells that were submitted to radiation alone, to cytotoxic autophagy in cells submitted to radiosensitization combined to vitamin D3 [62,63]. Additionally, in an independent study, vitamin D was shown to trigger Beclin1-mediated autophagic cell death in MCF-7 breast cancer cells [64]. 

In the same line, the recent work of Sheng et al. showed that the estrogen receptor (ER) status in breast cancer cells influenced the gemcitabine efficacy: ER expression promoted cytotoxic autophagy through the enforced activation of the ER-ERK-p62/SQSTM1 pathway, whereas ER negative cells underwent cytoprotective autophagy [65].

Similarly, a growing number of studies highlight that although the inhibition of EGFR signaling in non-small cell lung cancer cells induced cytoprotective autophagy at first, its further activation (through the addition of rapamycin [66] or through longer hypoxia exposure [67]), led to a switch towards autophagic cell death [68].

Other reports point out the importance of the p53 status (wild-type or mutated) in controlling the survival or death outcomes of autophagy [69]. Likewise, the inhibition of autophagy in colon cancer cells was shown to sensitize wild-type p53 cells, but not mutant p53 cells, to topotecan treatment [70]. In this model, the autophagy-mediated degradation of mutant p53 could possibly restore the onco-suppressive function of wild-type p53 and lead to tumor cell demise, as proposed by de Vries et al. [71]. 

The control of autophagy intensity by the anti-apoptotic Bcl2 family of proteins has also been abundantly studied. The literature in this field identifies a “Beclin1/Bcl2 rheostat”, acting in the control of cell survival and death decisions. The Bcl-2 and Bcl-xL proteins inhibit autophagy by binding to Beclin1 [72,73]. The disruption of these interactions increases the level of free Beclin1, which can subsequently strongly activate the autophagy process. Thus, the combination of the molecular depletion of Bcl-2 (through siRNA) with chemotherapy in breast cancer cells [74], and with nutrient starvation in neuroblastoma [75], was shown to potentiate autophagy and to promote massive tumor cell death. Still, in this framework, Lamy et al. reported in multiple myeloma that caspase-10 inactivation (using Q-AEVD-OPH or shRNA targeting caspase-10) led to the stabilization of the BCLAF1/Bcl-2 complex and the unleashed activation of Beclin1, which is responsible for the autophagy process over-activation, culminating in autophagy-mediated cell death [76].

The orientation towards an autophagy-mediated cell survival or cell death was shown to be controlled also by sphingolipid rheostat as well [77], i.e., by the balance between ceramide and sphingosine-1-phosphate levels [78]. Indeed, Scarlatti et al. reported that ceramide-induced autophagy triggered autophagic cell death [79], and Lavieu et al. demonstrated that sphingosine-1-phosphate induced survival autophagy [80].

Finally, different doses of a therapeutic compound could also trigger autophagy, but with opposite outcomes in a same cell line. In this context, Willems et al. reported that Acute Myeloid Leukemia (AML) cells treated with either low or high doses of the mTORC1 catalytic inhibitor (AZD8055) undergo autophagy with cytotoxic or cytoprotective functions, respectively [81]. Consequently, the authors suggest that combining chemotherapy (which induces cytoprotective autophagy) with low-dose AZD8055, or conversely, combining high-dose AZD8055 with autophagy inhibitors, may represent new strategies for improving AML treatment.

### 3.3. Other Therapeutic Use of Autophagy And Autophagosomes

#### 3.3.1. Oncogene Degradation

It has been demonstrated that several fusion oncogenes, notably PML-RARα in Acute Promyelocytic Leukemia (APL) [82], BCR-ABL in Chronic Myelogenous Leukemia (CML) [83], and FLT3-ITD in Acute Myeloid Leukemia (AML) [84] can be degraded through autophagy induced by specific treatment, i.e., arsenic trioxide or all-*trans* retinoic acid in APL; arsenic trioxide in CML and an inhibitor of the proteasome known to induce autophagy, the bortezomib, in AML. In these models, confocal colocalization experiments suggested the recruitment of the fusion oncogenes to autophagosomes via their interactions either with the p62/SQSTM1 (Sequestosome-1) or with LC3B proteins or both. Since these leukemic cells are known to be addicted to their leading oncogene, autophagy activation could be therapeutically exploited to force oncogene degradation and subsequently to lead to tumor regression. In this context, autophagy-mediated oncogene degradation contributes to the cytotoxicity of the drug. Interestingly, another leukemia-associated fusion oncogene, AML1-ETO, has not been found to be associated with autophagosomal vesicules [85]. The nature of the signals, which do or do not drive this autophagosomal localization, is not yet known. Also, the presence of the fusion oncogenes BCR-ABL and FLT3-ITD in the autophagosomes from resting cells (no therapeutic treatment) has not yet been reported. Finally, the localization of NPM-ALK in autophagosomes, in untreated and crizotinib-treated ALCL cell lines, is currently under investigation in our laboratory.

#### 3.3.2. Autophagosomes, As Carriers for Vaccination

A growing body of evidence demonstrates that autophagy has important roles in immunity [86], and that it could be used to improve cancer immunotherapies [59]. First, as previously discussed, autophagy is required for the immunogenic release of adenosine triphosphate (ATP) from dying tumor cells (upon chemo- or radio-therapy), which promotes the recruitment and activation of immune system effectors (dendritic cells and T lymphocytes) to trigger immunogenic cell death [87,88,89]. Second, the autophagy process, in antigen-presenting cells (APCs), was shown to participate in tumor antigen processing and presentation through both Major Histocompatibility Complex (MHC) class I and II molecules [90,91]. 

Interestingly, dendritic cell vaccines are considered to be a very promising anti-cancer vaccine strategy [92,93,94]. These vaccines involve the isolation of the patient’s dendritic cells (DCs), followed by ex vivo loading with tumor associated antigens (TAAs) in the presence of maturation stimuli, and subsequent re-introduction to the same patient. The loading of DCs with TAAs ex vivo can be achieved in many ways [93,95]; one of them being by incubating DCs with tumor-derived autophagosomes (also called DRibbles) [96,97]. Indeed, autophagosome accumulation in cancer cells, followed by their exocytosis, has been shown to be a superior preparation, from which DCs can “upload” tumor antigen for T-cell priming [98]. The efficiency of such DC-based autophagosomal vaccines has been tested in pre-clinical studies using melanoma, lung, and head and neck cancer models [96,99]. These findings could broaden the therapeutic use of autophagy in cancer cells for the development of cancer vaccines [100]. 

## 4. ALK-associated Cancers

ALK is a tyrosine kinase receptor which physiological expression is restrained to few neuronal cells during embryonic development [1]. Its aberrant oncogenic activity results either from ALK gene amplification, mutations, or chromosomal rearrangements [2,3]. A growing spectrum of cancers has been associated to the ALK oncogene, which has boosted the research towards ALK tyrosine kinase inhibition [7,101]. For the purpose of this review, we will focus our discussion on the ALK-associated cancers for which autophagy has been found to be activated in response to therapy. These main studies are listed in Table 1. Of note, Takeuchi et al. identified the fusion SQSTM1-ALK in ALK-positive large B-cell lymphoma, but the impact of this rearrangement on autophagy was not investigated [102].

### 4.1. Neuroblastoma

Neuroblastoma (NB) is the most frequent extracranial cancer in childhood (below five years of age), and it accounts for 15% of pediatric cancer deaths [110]. It is a heterogeneous disease, which can regress spontaneously or oppositely, which can be highly metastatic and resistant to treatment. This high risk subgroup is genetically complex. *MYCN* gene amplification and hemizygous deletions of 1p and 11q were found to be highly recurrent [111,112]. Regarding mutations, a few frequently mutated genes, including *ALK* (notably on residues R1275, F1174 and F1245), *PTPN11*, *ATRX*, *MYCN*, and *NRAS*, were identified [113,114]. ALK transcripts and ALK protein expression in NB were first described by Lamant et al. [115]. Germline mutations in ALK have also been shown to drive tumorigenesis in most cases of hereditary NB [116]. Thus, as ALK mutations, as well as amplifications [117], are found in NB pathogenesis, this tyrosine kinase is considered to be a major oncogenic driver of the disease and triggered the study of ALK inhibition as a therapeutic option [15,118]. However, the use of ALK inhibitors appeared to be quite inefficient in blocking NB growth, because of the emergence of drug resistance [119]. Recently, Aveic et al. showed that the activation of autophagy upon ALK inactivation (Entrectinib treatment), especially in ALK-mutated SH-SY5Y cells (harboring the F1174L mutation), was responsible for the low efficiency of the drug [105]. Autophagy induction and autophagic flux activation were monitored by LC3 turnover assay, in the presence or absence of Chloroquine (CQ) (used as a classical inhibitor of lysosomal degradation); by the degradation of the autophagy receptor p62/SQSTM1; and, by the increased formation of GFP-LC3 puncta when comparing Entrectinib-treated cells with untreated cells. The authors further showed that autophagy inhibition, using CQ, increased Entrectinib-induced cell death, as measured by both PARP protein cleavage and TUNEL assay. Altogether, this study identified a cytoprotective role for autophagy in Entrectinib-treated ALK-mutated neuroblastoma cells, and the authors suggest combining the pharmacological inhibition of autophagy with ALK inhibitor as a new possible approach to prevent drug resistance, and consequently reduce NB tumors recurrence and metastasis. 

Concerning NB harboring either *ALK* amplifications or *ALK* wild-type gene, the use of ALK inhibitors was shown to induce a strong proliferative arrest and a concomitant apoptosis and autophagic response [105,106]. In the latter case, it has been suggested that autophagy occurring following ALK inhibition had a synergistic deleterious effect with apoptosis [106]. Of note, it is likely that such a therapeutic synergy benefit also takes place in ALK-mutated neuroblastoma, when combining PI3K/AKT/mTOR inhibition (using Rapamycin) and Crizotinib [120].

Altogether, these studies interestingly suggest that depending on the *ALK* gene status, the autophagy that is activated following ALK inhibition could lead to opposite outcomes, i.e., cytoprotection in ALK-mutated neuroblastoma or cytotoxicity in ALK amplified or wild-type NB. Furthermore, in accordance with the notion that a sustained and unrestrained activation of autophagy could support its functional switch from cytoprotection to cytotoxicity (see paragraph “Autophagic switch”), the combined treatment of ALK-mutated NB with Crizotinib and a strong inducer of autophagy (Rapamycin) was reported to enhance tumor cell death and to be beneficial for patients [120]. 

### 4.2. Rhabdomyosarcoma

Rhabdomyosarcomas (RMSs) represent the most frequent malignant soft tissue sarcomas in childhood and young adulthood [121]. Two distinct subtypes have been described: alveolar rhabdomyosarcoma (ARMS) and embryonal rhabdomyosarcoma (ERMS), which account for 30% and 70% of RMSs, respectively. RMSs are currently treated with surgery and chemo- or radio-therapies, but resistances to these last treatments lead to a low rate of patient’ survival [122]. Aberrant ALK overexpression has been found predominantly in ARMS, but was also associated with disease progression in ERMS [123,124,107]. Thus, the impact of ALK inhibition on the biology of RMS cell lines has been studied recently. Megiorni et al. found that high doses of Crizotinib treatment impaired cell growth (blockade in G2/M phase) and induced apoptosis (caspase-3 activation/PARP proteolytic cleavage) [107]. Features of autophagy activation were also reported, such as a Crizotinib dose-dependent (i) increased Acidic Vesicular Organelles (AVOs) formation, (ii) increased conversion of LC3-I to LC3-II, with (iii) a concomitant down-regulation of p62/SQSTM1. The authors suggest that this autophagy activation may rely on an increased ROS production upon Crizotinib treatment, and that it could represent an alternative cell death mechanism. 

### 4.3. Glioblastoma

Glioblastoma multiformes (GBM) are the most lethal and most common malignant brain tumors in adults [125]. It is a highly hypoxic tumor, which is resistant to conventional chemotherapy or radiotherapy, and of poor prognosis [126]. ALK overexpression, without *ALK* gene amplification or translocation, has been shown in human tumors [127] and in glioblastoma stem cell lines (GSCs) [128], and ALK signaling has been associated with GSC self-renewal and tumor survival [129]. Recently, ALK signaling was shown to confer GBMs proliferative advantages through neovascularization [130]. ALK was shown also to prevent autophagy-associated cell death following cannabinoid (Δ(9)-Tetrahydrocannabinol (THC)) treatment [108]. Indeed, the authors demonstrated that the inhibition of ALK (by using ALK targeted siRNAs or the NVP-TAE-684 pharmacological inhibitor) enhanced THC-induced autophagy and subsequent apoptotic cell death [109]. Importantly, several studies highlight that, on the contrary, autophagy may serve survival functions under other therapeutic modalities for GBMs [131,132]. Further investigations are still needed to understand the autophagic response and to modulate it in the proper way (inhibition or activation) to improve GBMs therapies [126,133].

### 4.4. Anaplastic Large Cell Lymphoma

ALK-positive ALCL account for 10% to 15% of pediatric lymphoma [134]. They are characterized by two main chromosomal translocations: t(2;5) and t(1;2), leading, respectively, to the production of the NPM-ALK (70% of the cases) [135] and TPM3-ALK (18% of the cases) [136] fusion proteins. Their ongoing treatment, based on aggressive chemotherapy, is not optimal since tumor relapses are invariably observed in 30% of patients [134,137]. ALK tyrosine kinase-targeted therapies (using Crizotinib and further generation of ALK-TKIs) are currently used as second line therapy for relapse or refractory patients [16]. However, resistances to these ALK TKIs have been reported, both in ALK-positive ALCL cell lines and in patients [16]. Therefore, there is still a need to improve ALK-positive ALCL therapeutic treatment. As a multi-faceted approach to eradicate tumor cells, combined therapies for ALK-positive ALCL are currently being studied [18]. In this framework, our group is interested in understanding the role of autophagy in ALK-positive lymphoma therapy and how the autophagy process could be exploited to improve patients’ treatment. Our work showed that autophagy was induced in ALK-positive ALCL cell lines following pharmacological (Crizotinib) or molecular inactivation (through ALK-targeted siRNA) of NPM-ALK [103]. This was assessed by a combination of complementary approaches: increased AVOs formation, increased number of degradative autophagic vacuoles (as detected by electron microscopy), and increased LC3-II dot staining (as revealed by immunohistochemistry performed on paraffin embedded Karpas-299 cells). Moreover, autophagic flux activation was demonstrated by the classical LC3 turnover assay using either Chloroquine or siRNA targeting ATG7 as inhibitors of the autophagy degradation process. We further demonstrated, both in vitro (by performing viability, apoptosis and clonogenic assays) and in vivo (by measuring xenografted tumor growth), that the combination of autophagy and ALK inhibitions led to the potentiation of the targeted therapy, thus highlighting the cytoprotective function of autophagy in these settings [103].

We are pursuing this work by investigating whether we could induce an autophagic switch from cytoprotection to cytotoxicity, in Crizotinib-treated cells. We are interested by the potential involvement of BCL-2 in the regulation of this switch [138], as previously reported in other models (see Figure 2). We also identified a microRNA whose overexpression potentiated the Crizotinib-induced autophagic flux and decreased the cell viability [139]. Thus, our current data indicate possibilities to balance from cytoprotective to cytotoxic autophagy in Crizotinib-treated ALK-positive ALCL cells. 

### 4.5. Non-Small Cell Lung Carcinoma

Lung carcinoma is the most common cause of cancer-related deaths, with a five-year survival rate of 15%. Clinically, they are divided into two subgroups: NSCLC (85–90%), and small-cell lung cancer (SCLC) (10–15%). Surgery is the optimal treatment for non-metastatic NSCLC, which are less sensitive to chemo- and radio-therapies than SCLC. *ALK* rearrangements occur in around 4–5% of NSCLC cases, the predominant one being the inversion in the short arm of chromosome 2, which creates a fusion between the echinoderm microtubule-associated protein-like 4 (*EML4*) gene and the *ALK* gene, leading to the production of the EML4-ALK fusion protein [3,140,141]. Due to the urgent need for therapies in this subset of patients, Crizotinib was rapidly evaluated [11,142], and has been approved as first line therapy in ALK-positive NSCLC [143]. However, many resistance mechanisms (mainly through ALK mutations, ALK amplification or the activation of compensatory signaling pathways (EGFR upregulation; KRAS mutations)) have been described [144,145], which highlight the continuous necessity for improved therapy. In this context of drug resistance, Ji et al. reported that autophagy was activated in H3122 Crizotinib-resistant cell lines following high dose treatment (as shown in LC3 turnover assay), and its inhibition (by using Chloroquine or Bafilomycin A1) allowed an increased sensitivity to the drug, both in vitro (as shown by reduced viability and clonogenic potential, as well as increased Annexin V positive cells in apoptosis flow cytometry analysis) and in vivo (reduced xenografed tumor growth) [104]. However, since EML4-ALK expression was not detectable in these cells, it is likely that the high dose of Crizotinib that was used in this study (up to 8 μM) in fact induced cytoprotective autophagy, but independently of EML4-ALK inactivation. The authors finally propose to modulate autophagy in order to circumvent Crizotinib resistance in ALK-positive lung cancer, but the extent of these findings in clinical settings is not yet known. 

## 5. Conclusions

In literature, autophagy has been shown to be induced upon therapies in different kinds of ALK-associated cancers. Further investigations are globally necessary to better characterize this autophagic response upon treatment. Notably, the molecular pathways that are responsible for sensing the therapeutic stress and its relation with autophagy induction in ALK-related cancer cells, still remain to be clarified. Most importantly, as therapeutically-induced autophagy can drive tumor cells’ fate towards survival, or oppositely, towards death, the identification of the actors and regulators of this switch should provide more effective ALK-cancers therapies. The development of drugs, which specifically inhibit or activate the autophagic process [146], and the search for the “right” therapeutic combinations, which could promote the appropriate autophagic response (i.e., cytotoxicity) are expanding research fields. To benefit from these advances, it is important to first investigate, when possible, the autophagic status in a patient’s tumor. Throughout these last years, great progress has been made in the measure of the relative abundance of key markers of the autophagy process (LC3 and p62/SQSTM1), by immunohistochemistry. This technique could be used to monitor the autophagy dynamics in paraffin-fixed tumor tissues [147]. The search for autophagy gene abnormalities (amplification, deletion, mutations) in ALK-associated cancers would also bring useful information on the status of autophagy in primary and/or relapsed ALK-associated tumors. The development of biomarkers of the autophagic status in bodily fluids of cancer patients could also help at diagnosis, and could potentially orient the therapeutic strategy [148]. Finally, in regards of the complex, yet very important role of autophagy in cancer therapy, an approach that is worth considering would be the use of autophagy modulation in Patient-Derived Xenograft models. This kind of model has already been successfully developed for ALK-positive ALCL [149,150,151], ALK-positive NSCLC [152], Rhabdomyosarcoma [153], and Neuroblastoma [154], and are considered as the most reliable tumor model. This approach could help to decipher the importance of autophagy in ALK tumorigenesis, and also to develop new ways to improve ALK-associated cancer personalized therapies.

## Figures and Tables

**Table:** 

Cancer model	Condition 1	Condition 2	Reference
Breast cancer	Radiotherapy	Radiotherapy + vitamin B3	[62,63]
Breast cancer	Gemcitabine	Gemcitabine + Estrogen receptor signaling	[65]
Non Small Cell Lung cancer	Low inhibition of EGFR signaling	High inhibition of EGFR signaling	[66,67]
Colon cancer	Topotecan	Topotecan + mutated p53 status	[70]
Breast cancer Neuroblastoma Multiple myeloma	Beclin 1/Bcl-2 complex	Disruption of Beclin 1/Bcl-2 complex	[74] [75] [76]
Breast cancer Colon cancer	Sphingosine 1 phosphate signaling	Ceramide signaling	[79] [80]
Acute myeloid leukemia	Strong mTOR inhibition	Low mTOR inhibition	[81]

**Table 1:** Studies reporting autophagy induction following therapy in Anaplastic Lymphoma Kinase (ALK)-associated cancers.

Cancer Type	*ALK* Gene Aberration	Treatment	Method(s) Used to Monitor Autophagy	Role of Autophagy	Signaling Pathway Involved	Refs
ALK+ALCL	Translocation (mainly NPM-ALK)	Crizotinib	Electron microscopy Western Blotting Immunohistochemistry Autophagy array (qPCR) Acridine Orange	Cytoprotective	Akt-mTOR suggested	[103]
ALK+NSCLC	Translocation (mainly EML4-ALK)	Crizotinib	Western blotting Electron microscopy	Cytoprotective	Akt-mTOR	[104]
NB	Mutations Amplification	Entrectinib AZD3463	Western blotting	Cytoprotective Cytotoxic role suggested	Not studied PI3K/Akt/mTOR	[105] [106]
ARMS	Gain in copy number	Crizotinib	Acridine Orange Western blotting	Cytotoxic role suggested	PI3K/Akt/mTOR suggested	[107]
GBM	No aberration reported	THC	Immunohistochemistry Western blotting	Cytotoxic	Midkine through Akt/mTORC1	[108] [109]
